# Are the Claims to Blame? A Qualitative Study to Understand the Effects of Nutrition and Health Claims on Perceptions and Consumption of Food

**DOI:** 10.3390/nu11092058

**Published:** 2019-09-02

**Authors:** Tony Benson, Fiona Lavelle, Amanda McCloat, Elaine Mooney, Tamara Bucher, Bernadette Egan, Moira Dean

**Affiliations:** 1 Institute for Global Food Security, School of Biological Sciences, Queen’s University Belfast, Belfast BT9 5AG, UK; 2 Department of Home Economics, St. Angela’s College, Sligo F91 C634, Ireland; 3 School of Health Sciences, Faculty of Health and Medicine, The University of Newcastle, Callaghan, NSW 2308, Australia; 4 Priority Research Centre for Physical Activity and Nutrition, The University of Newcastle, Callaghan, NSW 2308, Australia; 5 Food, Consumer Behaviour and Health Research Centre, University of Surrey, Guildford GU2 7XH, UK

**Keywords:** nutrition claims, health claims, portion size, perceptions, health halo, food labelling, consumer, nudging, qualitative, focus groups

## Abstract

Nutrition and Health Claims (NHCs) have been found to influence perceptions of food and consumption behaviour. While previous quantitative research has identified factors that may explain these effects, the current study aimed to address the dearth of in-depth exploration as to the underlying reasons why and how claims may impact upon perceptions and behaviour and the relationships between key factors. Seventy-eight participants took part in 10 focus groups. Discussions were transcribed verbatim and Nvivo 11 was used for thematic analysis. Six themes were developed from the data: 1. Target populations for NHCs; 2. Influence of NHCs on purchasing behaviour; 3. Characteristics/perceptions of products displaying NHCs; 4. Believability of NHCs; 5. Superior yet superficial knowledge; 6. Consumption of products displaying NHCs. Knowledge was a key factor influencing how much individuals believe claims (Believability of NHCs) and their perceptions (Characteristics/perceptions of products displaying NHCs). These perceptions and the characteristics of products displaying claims also impacted believability, as well as purchasing behaviour and consumption. Future research should be cognisant of the role of knowledge and characteristics or perceptions of products in the relationship between NHCs and consumer behaviour, and modelling of these relationships would allow their relative strength to be identified.

## 1. Introduction

Nutrition and health claims (NHCs) are text or images on food or drinks products which highlight their nutritional or energy content and (for health claims) their impact on health. Specifically, a nutrition claim is a claim on a food or drink product which indicates that a food has beneficial nutritional properties due to the energy it provides or does not provide or the nutrients or other substances it contains or does not contain [1], for example “low in fat”. A health claim is a statement that suggests or implies that a relationship exists between food and health [1] such as “… contains calcium which helps maintain healthy bones”.

Nutrition and health claims may help consumers to make informed decisions about their purchase and intake of food and drink, which in turn may lead to an improvement in diet quality [2]. However, it has also been suggested (somewhat paradoxically) that NHCs may also lead to an increase in consumption, with a recent systematic review and meta-analysis finding that NHCs increase consumption and/or purchasing behaviour of food and drink [3]. The increase in consumption associated with NHCs may be due to a “health halo” effect [4] whereby an individual generalises from a nutrition or health claim that a product is healthier or has more favourable attributes than it actually does. NHCs may also impact consumers’ perceptions of food products, with several studies showing impacts on perceived healthiness, tastiness, fillingness, naturalness, and attractiveness [5,6,7,8]. Specifically, claims have been found to increase perceived healthiness, decrease tastiness, attractiveness and naturalness perceptions and both increase or decrease fillingness perceptions, depending on the product. This research is further supported by a recent systematic review which found that overall, although based on low quality evidence, NHCs did influence consumers’ perceptions of products [9].

The use of nutrition and health claims varies across countries due to cultural differences in regulation and understanding [10,11]. For example, 50% of individuals in Sweden check nutrition information on labels at least occasionally compared with 65% of individuals in Ireland. Specifically in relation to nutrition and health claims, a supermarket audit in Ireland found prevalence rates of 47% for nutrition claims and 18% for health claims [12]. This compares with European rates of 21% and 11% respectively [13] and an Australian rate of 14% for any claim [14]. While these studies are not directly comparable due to differences in methods and years of study (and interim changes in legislation, such as the establishment of the European Union (EU) register of nutrition and health claims), these results suggest that Ireland has a relatively high prevalence of NHCs. Despite these cross-country differences and the introduction of regulations in this area over 10 years ago, little research examining nutrition and health claims has been conducted on the island of Ireland. 

A plethora of studies have investigated NHCs, yet scope for further research remains. For example, recent systematic reviews have highlighted the need for further studies on the effects of claims on dietary choices and consumption [3,9]. While the field consists mainly of quantitative studies, qualitative methods have also been used to examine NHCs. For example, a study using focus groups in the USA found that NHCs increased parents’ healthiness perceptions of children’s foods [15]. Similarly, Chan, Patch, and Williams [16] found that some Australians admitted that the presence of a low or reduced fat claim increased their consumption. Other focus group studies have found that claims could influence older women to purchase carrier products in Ireland [17], general acceptance of the potential for enhanced flavonoid content in fruit [18] and trust/distrust and relevance of claims as key themes [19]. While the outlined studies provide useful information on consumer attitudes towards and processing of claims, qualitative studies in this area have tended to investigate consumers’ general perceptions and understanding of claims rather than the influence of claims on perceptions and consumption. From these studies, several common findings have emerged, showing that consumers lack understanding of some claims, NHCs may influence consumption and purchasing behaviours, and that consumers can be skeptical about claims. However, less well known is the structure and relationships between these factors and how NHCs influence consumer perceptions and behaviour. What are the pathways or underlying mechanisms by which claims influence the consumer? It is known that consumer characteristics such as sociodemographics and knowledge play a role in the link between claims and behaviour [3,5], but less well known is the relationship between these factors and their possible interactions and how these might mediate the effects of claims on perceptions and consumption behaviour. For example, it is known that NHCs impact consumption [3] but less attention has been given to why this happens or the psychological explanations for such effects. Therefore, these pathways should be explored.

The current research aimed to address the aforementioned limitations by using qualitative research to understand the relationships and pathways between NHCs and consumers’ attitudes and behaviours and understand how and why NHCs influence perceptions and consumption behaviour. The current study also built upon the limited amount of previous qualitative research in this area conducted on the island of Ireland (includes Northern Ireland (NI) and the Republic of Ireland (ROI)) by using a mixed sample (in contrast to a young female sample previously used).

## 2. Materials and Methods 

Ethical approval (07/16/BensonT) was granted by the Queen’s University Belfast School of Biological Sciences Research Ethics Committee and written informed consent was obtained from all participants. The study was conducted in accordance with the Declaration of Helsinki.

### 2.1. Participants

Participants were recruited using convenience and snowball sampling. Given their potential to bias the results, individuals who were currently studying or had ever undertaken a degree in food, nutrition, or dietetics were excluded from participation. Similarly, those (or those with anyone in their household) working in any of the following areas were also excluded: advertising, marketing or market research, the food industry, diet, or nutrition. In total, 78 participants (see Table 1 for characteristics of participants) were recruited to ten focus groups (five held at a location in NI, five held at a location in ROI). Given the subject matter and the fact that the presence of the opposite sex can influence eating behaviours [20,21], groups were single sex (five exclusively male groups and five exclusively female groups), consisting of between six and nine participants and either younger (18–35 years old) or older (36–64 years old) with an overall mean age of 36.6 (SD = 13.2) years.

### 2.2. Materials

#### 2.2.1. Elicitation Materials

Several products and product images displaying NHCs were used as prompts in the discussion (see Table 2 for an overview of the materials used). To assess the spontaneous awareness and recognition of NHCs, participants were shown a chocolate bar displaying the regulated nutrition claim “no added sugar”, as well as phrases which may be perceived as NHCs, for example, “gluten & nut free”. This bar was also chosen as it was classified as “less healthy” according to the Office of Communications (Ofcom) nutrient profiling system [22]. Furthermore, recent research has called for examination of the effects of claims on hedonic products [23]. Therefore, it was felt that it would be interesting to gauge participants’ opinions of the apparent juxtaposition of a “less healthy” product (typically viewed as a treat food) displaying nutrition and health messages. 

The most common nutrition and health claims in the UK and Ireland relate to fat [12,24]. Therefore, the second product used as a prompt was breakfast cereal labelled with the claim “low in fat” as well as “source of vitamin D”. A still image from a yoghurt advertisement was also used as a discussion prompt. This contained the health claim “… contains calcium which helps maintain healthy bones”.

#### 2.2.2. Questionnaire

Participants completed a short questionnaire (approximately ten minutes in duration) which collected sociodemographic information, such as age, sex, education, and socioeconomic status. Socioeconomic status was categorised as higher (ABC1—higher and intermediate managerial or professional occupations) or lower (C2DE—unskilled, semi-skilled, skilled occupations and those unemployed). Other factors which may influence perceptions of nutrition and health claims [25] were also assessed, such as current dieting and health issues and motivation to process (use of) nutrition and health claims [26].

### 2.3. Procedure

Following a review of the relevant literature, a semi-structured topic guide was created to explore attitudes towards and perceptions of nutrition and health claims. This was piloted with a group of eight female participants, with subsequent minor amendments made prior to full implementation, such as the addition of show cards with the definitions of “nutrition claim” and “health claim”. Each focus group discussion was guided by an experienced moderator (TB) using the final topic guide (see Table 3). The groups began by outlining the purpose of the group and use of recording equipment. The order of the topic guide was designed to allow spontaneous mentions of NHC, followed by specific questions focusing on perceptions (tastiness, healthiness, and satiety) and consumption behaviour in relation to NHCs. Participants were also asked about their familiarity with and knowledge of NHC. Following the discussion, participants completed a questionnaire (see Section 2.2.2 Questionnaire). Each group session was audio recorded and lasted approximately 60 minutes. Upon completion, participants were paid an honorarium of £20 (NI) or €25 (ROI) to compensate for time and travel costs. 

### 2.4. Data Analysis

Audio recordings from the groups were professionally transcribed and imported into NVivo 11 (QSR International Pty Ltd, Doncaster, Victoria, Australia) for analysis. Inductive thematic analysis was used to interpret the data, following the procedures of Braun and Clarke [27]. All the transcripts were read at least twice (by TB, psychologist in health) to achieve familiarity with and immersion in the data, with any prominent concepts in the data noted. Initial codes in the data were developed and subsequently arranged into themes. These themes were reviewed and checked by a second researcher (FL, a sport and health scientist) and any necessary refinements were made. Following this, the themes were named and defined and any links or relationships were established. Finally, transcripts were read again to ensure that the themes accurately represented the data and that there were no further applicable themes. Appropriate quotations were then chosen to represent each theme.

## 3. Results

Six themes were developed from the analysis: 1) Target populations for NHCs; 2) Influence of NHCs on purchasing behaviours; 3) Characteristics/perceptions of products displaying NHCs; 4) Believability of NHCs; 5) Superior yet superficial knowledge; 6) Consumption of products displaying NHCs. The meaning and content of these themes are explored in more detail below, with relevant quotations provided.

### 3.1. Target Populations for NHCs

There was general recognition amongst the groups that certain types of individuals or groups were most likely to benefit from using products with NHCs. It was commonly mentioned that those on diets or managing their weight, as well as those eating healthily, might be more likely to use these products:

“Yeah, I just joined Slimming World, so that’s a big thing for me. I’m looking for 0% fat on things like dairy products, fromage frais, that kind of thing. Or, Diet Coke, I’d go to that, because it’s no sugar compared to Coke…”

(Group 4, female, 18–35 years old)

Those with specific illnesses or conditions were also mentioned as possible target populations for products with NHCs. In addition, parents were identified as purchasing products with claims for their children:

“…you do want them (children) to grow up and they need healthy bones so the parents would get the stuff that contains calcium.”

(Group 10, male, 18–35 years old)

### 3.2. Influence of NHCs on Purchasing Behaviour

Participants recognised that NHCs could sometimes influence their purchasing behaviours such that claims encouraged them to buy a product:

“…that’s a big factor, isn’t it, you know, saying “diet” on it or “light”. You would think ’okay I’ll go for that option.”

(Group 1, male, 36–64 years old)

In contrast, other participants remarked that claims did not influence their purchasing behaviour and that they did not look for these when shopping:

“… I wouldn’t purposely go out and say ‘I’m only buying yogurts that are fortified with calcium…’”

(Group 7, female, 18–35 years old)

When probed as to when or how NHCs might influence purchase, a subset of those who acknowledged that their purchasing behaviour might be influenced mentioned the type of product or the brand was a factor:

“A lot of the breakfast choices I’m influenced, like I would eat oats because I’m influenced by the cholesterol thing (claim)…”

(Group 2, female, 36–64 years old)

There was also some acknowledgement that while NHC on packaging may not directly impact upon purchasing, claims sometimes made them more interested in a product and invited closer inspection.

### 3.3. Characteristics/Perceptions of Products Displaying NHCs

The presence of a nutrition or health claim on a product affects consumer perceptions of the product. Participants mentioned that there was often a price premium to be paid for the apparent benefits that come from consuming a product displaying claims:

“They make it more expensive, I suppose. They put up the price of it… making it that bit more expensive than the competitors.”

(Group 10, male, 18–35 years old)

Participants also understood the presence of an NHC to mean that ingredients had been substituted in order to achieve the claim. The example of a low-fat claim meaning that the reduction in fat had led to an increase in sugar was commonly cited. Somewhat linked to this substitution of ingredients, it was commented that this could lead to a change in the taste of products. Participants particularly mentioned that products with NHCs might be bland or poor in taste:

“You know, you just see "low in fat" and you think it’s missing something, something’s been pulled out of the process and it’s not going to taste as good.”

(Group 6, male 36–64 years old)

Also linked to the substitution of ingredients, a few participants mentioned that products with claims may be unhealthy, for example due to being higher in sugar. However, overall, participants viewed products with NHCs as being healthier than products without claims:

“Yes, if something has a health claim, like anti-oxidants, things like that, you do tend to think of them as healthy things or good things.”

(Group 2, female, 36–64 years old)

Interestingly, the presence of claims on the health food shop chocolate bar seemed to confuse participants and prompted discussion as to whether the bar was ‘healthy’. Despite being classified as a less healthy food according to its nutrient profile score, a sizeable proportion of participants felt that the bar was healthy. This suggests that consumers’ typical healthiness perceptions of a food may be strongly influenced or changed by claims and that nutritionally less optimal products might be viewed as healthy. Finally, there was a perception amongst some participants that NHCs impact upon the portion size of products. Specifically, it was felt that products bearing NHCs have smaller serving and/or portion sizes in order to achieve their claims:

“…it says it’s low in fat but it weighs less than something beside it which doesn’t say low in fat.”

(Group 4, female, 18–35 years old)

### 3.4. Believability of NHCs

There were a number of different factors which impacted whether participants believed a claim or not. Overall, there was a strong awareness that NHCs are controlled by law, either in terms of regulations or with regards to advertising standards. This led to a general belief that claims must be true:

“The only reason I would believe it is because if it’s on TV there’s regulations, so it has to be true.”

(Group 3, male, 18–35 years old)

Despite this awareness of regulations, consumers were still skeptical:

“I would generally be naïve enough to believe it, yes. I would take them at their face value, because they should be basing it on the nutritional facts, but… how often do they check to make sure those facts are right?”

(Group 1, male, 36–64 years old)

The brand of a product was another factor which influenced the believability of NHCs:

“Before we would have tended to trust the big names, like McVitie’s or Kellogg’s, but now… we don’t really trust them and the whole thing has lost its credibility. It’s just a series of numbers and percentages and grams and claims and people generally don’t really trust them.”

(Group 2, female, 36–64 years old)

The type of product and its suitability for certain claims was mentioned with differing opinions. A few felt that claims on healthier products such as cereal were more believable than claims on less healthy products, while others felt that there should be a link between the claim and the ingredients for it to be believable. However, when the claim was ‘linked’ with the ingredients or type of product, some felt that this was stating the obvious:

“That’s kind of common sense, to me, like it’s a yoghurt, it’s made from …it came from cream, it comes from milk, you know what I mean?”

(Group 8, male, 18–35 years old)

The fact that some claims might be stating the obvious led to discussions around skepticism and whether NHCs are simply a marketing ploy designed to encourage consumers to buy products, or whether they are useful signposts to the consumer which highlight beneficial nutritional properties:

“There’s certain foods you eat and they stick these labels on, low in fat or low in sugar, but the food is naturally going to be low in fat. It’s just marketing, to make you think.”

(Group 1, male, 36–64 years old)

Some participants felt that claims were believable in that while they may not help everyone, they may help certain individuals such as the elderly or those with certain conditions.

### 3.5. Superior yet Superficial Knowledge 

Overall, participants were aware of NHCs and showed some general awareness and knowledge. When asked, participants were able to provide examples of claims and examples of the types of products on which claims were typically displayed. There was a basic understanding of what claims mean:

“It means that per 100 grams, or whatever the measure is, that there’s a certain level where... there’s categories, low, medium and high, it will be under the, obviously low, on the low spectrum to do with fat.”

(Group 1, male, 36–64 years old)

There was also some evidence of superiority bias, with participants feeling that their knowledge was better than others (the general public) and that they would not be ‘fooled’ by NHCs:

“Well, I hope somebody doesn’t eat one and says ‘oh God, that’s making my bones so much easier’ because you would have to do it over a matter of time, you’d have to maintain it.”

(Group 5, female, 36–64 years old)

Overall, however, the knowledge shown by participants was basic and superficial. All groups were asked for the exact meaning of the term “low in fat” and none of the participants in any of the groups were aware that this refers to a product having 3 g of fat or less per 100 g. Participants were also unable to distinguish between nutrition claims and health claims. When asked for examples of both separately, participants cited nutrition claims and health claims together as well as unofficial or unregulated claims, such as “gluten free” and “organic”. This poor knowledge may be linked to the feeling of confusion or overload that some participants experience:

“It’s almost like there is too much information and so many different claims, and then you hear these claims are false.”

(Group 2, female, 36–64 years old)

### 3.6. Consumption of Products Displaying NHCs 

As well as claims influencing purchasing behaviour, participants also recognised that NHCs can sometimes influence their consumption, with many stating that claims had or would increase their consumption. The claim on the product appeared to license further consumption:

“I think low calorie, as I was saying, the WeightWatchers thing, you end up having two or three packets, because you think it’s very low calorie.”

(Group 1, male, 36–64 years old)

There was also mention of compensating intake after eating a claim product, typically altering or increasing their intake later, having earlier eaten a product carrying NHCs:

“Those breakfast biscuits, because they are supposed to fill you, but an hour later you’re saying ’it was only a light one anyway, I’m grand,’ and you have a bowl of cereal, and then you have a slice of wholemeal bread because I’ve had low fat stuff this morning…”

(Group 7, female, 18–35 years old)

Perhaps somewhat linked to participants’ views that products with claims can taste bland or poor, a few participants mentioned that NHCs might decrease consumption due to the taste of products carrying these claims:

“The taste was just like ’I can’t eat this.’ It might be healthy but it’s rotten”

(Group 1, male, 36–64 years old)

## 4. Discussion

**Figure 1:**
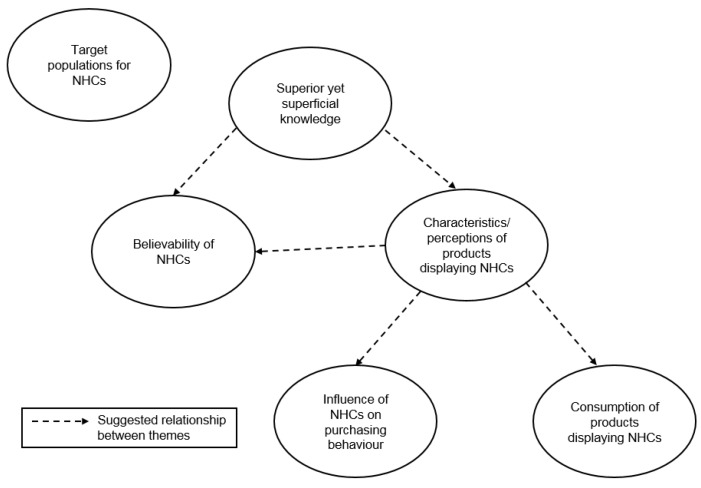
Relationships between constructed themes.

Numerous studies have investigated prevalence and understanding of and attitudes towards NHCs. It is generally well established that claims can influence consumer perceptions and consumption/purchasing behaviour of food and drink [3,9]. Specifically, claims are more likely to increase purchasing or consumption behaviour as well as increase healthiness perceptions of foods. It is also known that consumers may find NHCs useful but can also be confused and skeptical of their statements [15,16]. Less attention has been paid to the links between these findings and the possible underlying reasons why claims can impact the consumer. Using a qualitative approach, the current study aimed to identify and examine these links and reasoning. Six key themes were developed using thematic analysis: Target populations for NHCs, Influence of NHCs on purchasing behaviours, Characteristics/perceptions of products displaying NHCs, Believability of NHCs, Superior yet superficial knowledge, and Consumption of products displaying NHCs. While the themes were distinct, potential relationships between the themes were apparent, as displayed in Figure 1 and discussed in more detail below. 

The ‘Target populations for NHCs’ was a simple, standalone theme. Participants recognised that products displaying nutrition and health claims were likely to be most beneficial (perceived relevance) to individuals with certain health issues or conditions, or specific groups within the population. While some participants recognized themselves as being part of those who would benefit from products with claims (such as those trying to lose weight), in general, it was mentioned that other groups would benefit. This may be due to participants not having relevant health conditions or a reluctance to disclose health conditions in a group setting. In addition, some participants may not have identified as being part of the target group even though they fit the profile of the target group in terms of age, parenthood, and those attempting weight management. Pregnant women, the elderly, those with diabetes, and those attempting to lose weight or gain muscle were all mentioned as potential key users of products with claims. 

Participants’ knowledge of NHCs appeared to be limited, lacking depth. While it is already known that nutrition knowledge impacts upon perceptions of claims [23], fewer studies have specifically looked at NHC knowledge. One study found that nutrition and health claim knowledge may be relatively low [5]. The superiority bias shown in the current study (individual’s beliefs that they would not be misled by claims but others might) provides greater insight into an optimistic bias which has been found [5], whereby consumers believe that they have greater knowledge than others but actual knowledge is relatively low. Knowledge appeared to impact upon believability in the current study. If participants had knowledge or awareness of the ingredients, then they were more likely to believe the claim. For example, participants mentioned that they knew that calcium helps bones and therefore, a claim linking the two was believable and plausible. Lalor and colleagues [28] found that, overall, nutrition knowledge was not associated with perceived credibility of health claims, yet education level has been linked with belief in NHCs [29]. A link between knowledge and believability was seen in the current study as participants appeared unable to distinguish between different types of claims, for example, nutrition and health claims and unregulated and regulated claims. This may have impacted the believability; if participants are unaware that claims are regulated and there are stipulations for different claims, this may lead to low levels of belief and skepticism. Belief may also be linked with the wider concept of trust, which has previously been found to influence perceived benefits of claims [30]. In particular, those who have more trust in information sources tended to view claims as more beneficial than those with less trust, and food and health authorities were the most trusted sources. In addition, trust has been found to be a significant predictor of purchase intentions for functional foods (chocolate, yoghurt, and soup) [31]. Together, these findings might suggest that the type of knowledge, for example, knowledge in general, nutrition knowledge, or health knowledge is important as to whether knowledge influences believability. Future research should empirically investigate believability and different knowledge domains to determine their significance in the processing of claims.

Knowledge of NHCs (superior yet superficial knowledge) also had an impact on ‘Characteristics/perceptions of products carrying NHCs’. For example, a few participants noted that a claim means that the ingredients in a product have been affected, such as the substitution of fat with sugar. This also appears in the wider literature [32]. This relationship reflects the findings in the literature that knowledge influences the acceptance of products with NHCs. Bimbo and colleagues [33] found that a higher diet/health related knowledge was linked with greater acceptance of dairy functional products, while Drolias and colleagues [34] found that nutrition knowledge was associated with a reported use of health claims but not nutrition claims. 

The theme ‘Characteristics/perceptions of products carrying NHCs’ also has a relationship with the ‘Believability of NHCs’ theme. Current research in this area has identified participants’ belief in claims (believability) as a possible key factor or moderator of the relationship between NHCs and perceptions or behaviour [5,35]. However, unlike other studies in which believability has impacted upon perceptions of products [5,36], in the current study, belief was mentioned more in relation to skepticism, with little or no explicit mention of perceptions or consumption behaviour. This may be due to a true lack of connection between believability, perception, and behaviour, or participants simply not bringing these connections to mind in the focus groups. As previously mentioned, believability is a key factor which merits further investigation in relation to nutrition and health claims [5].

Nutrition and health claims influenced purchasing behaviour for some participants, and while others were not influenced, there was acknowledgement that NHCs captured their attention and led them to inspect a product. As a few participants stated that NHCs only influenced their purchasing behaviour for certain types of products, this theme was linked with the theme ‘characteristics/perception of products displaying NHCs’. In particular, breakfast and dairy products such as cereal, milk, and bread were mentioned as products where claims may be more likely to influence purchasing. This aligns with past research which has found that the type of product is perhaps one of the most important factors which affects consumer perceptions of products displaying NHCs [37,38]. For example, NHCs have been found to influence perceptions of cookies [8] but not those of cereal [39]. If certain types of products displaying NHCs are more likely to influence consumers, it may be necessary to ensure that only the products which are most nutritionally beneficial and suitable are authorised to display NHCs. Research in this area has examined the nutrient profile of foods carrying claims, finding that a sizeable proportion of foods with claims are not qualified to make a claim when subjected to a nutrient profiling system [40,41,42]. While Australia and New Zealand currently use a nutrient profiling system to approve claims on foods, such a system is yet to be established within the EU. The European Commission (EC) is currently conducting a re-evaluation to assess whether nutrient profiling for foods displaying NHCs is still necessary, the reasons preventing the introduction of profiles, and the costs and benefits of the absence of profiles [42]. Any creation of regulation in this area should be mindful of potential adverse effects. A recent study which modelled the potential impact of nutrient profile scoring in the UK found that while a nutrient profiling system might positively impact mortality rates if additional action is taken, such as the reformulation of products, the introduction of profiling without subsequent reformulation could lead to an increase in mortality rates as a result of less healthy diets [43]. However, as acknowledged, these are preliminary findings with large uncertainty intervals and further investigation is required. 

Finally, there was acknowledgement from participants that their consumption behaviour could or had been affected by claims. There was the potential for a licensing effect of claims, whereby individuals reasoned that they could eat (more) of a product, with claims that it is healthy [44]. However, this was typically discussed in the context of specific brands or types of food, such as breakfast bars, cereals, and crisps. Furthermore, some participants noted that the pack size of products displaying claims can be smaller in order to meet the requirements for a claim. For example, lower calorie products may have less calories compared with standard products due to containing less food rather than reformulation. While previous qualitative research found that participants ate additional packs to compensate for a reduction in pack size [32], to our knowledge, this has not been investigated in more detail. Empirical examination of the effects of claims controlling for pack size would be interesting to understand if a “health halo” effect still persisted. Together, these findings suggest a potential relationship between the themes ‘Consumption of products displaying NHCs’ and ‘Characteristics/perceptions of products displaying NHCs’. This is supported by a systematic review and meta-analysis of the effects of claims which found a preference for or consumption of foods with claims differed depending on the type of product [3]. As previously mentioned with regards to purchasing, if product characteristics also influence consumption behaviour, then it is important that the product has a beneficial nutritional profile and is suitable to display a claim. While several studies have examined the prevalence of food and drink products carrying claims and their nutrient profile [40,41,42], few have demonstrated the real-world implications of applying nutrient profiling to products with NHCs. Despite Australia having introduced a nutrient profile model for health claims, the impact of this does not appear to have been evaluated. Future work should examine or model the benefits and consequences (in areas such as public health and economics) of profiling products with claims, particularly as the development of an EU nutrient profiling model is pending.

Previous research in this area has identified key factors in the relationship between NHCs, perceptions, and behaviour. The current study advances research in this area by identifying the potential relationships between these factors and reasons underlying these pathways by using a qualitative approach. This investigation allowed for the creation of a model which can be taken forward in future research. The results suggest that knowledge (in particular familiarity and NHC knowledge) and characteristics/perceptions of products with NHCs are key influences on the purchasing and consumption of products displaying NHCs. The pathways and strength of the associations between factors should now be empirically validated. In addition, other factors from elsewhere in the literature and other potential relationships between factors which were not identified in the current study might also be tested. For example, there may be links between knowledge and target populations through perceived relevance if individuals believe that certain substances or nutrients identified by claims are most effective for certain population groups. A further strength of this study relates to the sample. While the use of a representative sample is unnecessary for qualitative research, this study used a relatively large and mixed group of participants, which allowed for a diverse range of views to be gathered. However, a greater proportion of participants were of a higher socioeconomic status and this should be considered in the interpretation of the results.

## 5. Conclusions

This study presents qualitative findings which provide further insight into the underlying reasons why nutrition and health claims may influence consumer perceptions and consumption behaviour. Knowledge was found to be a key influence on how claims are perceived. How much individuals believed claims appeared to be affected by the type and characteristics of products displaying claims. These characteristics alongside individuals’ perceptions of claims may in turn impact upon purchasing and consumption behaviours. 

## Figures and Tables

**Table 1:** Characteristics of focus group participants (*n* = 78).

Characteristic	*n* (%)
Location	*n =* 78
Northern Ireland	37 (47%)
Republic of Ireland	41 (53%)
Sex	
Male	38 (49%)
Female	40 (51%)
Education	
Primary school or less	1 (1%)
Secondary school (to age 15/16)	7 (9%)
Secondary school (to age 17/18)	16 (21%)
Additional training	13 (17%)
Undergraduate	17 (22%)
Postgraduate	24 (31%)
Socioeconomic status ^1^	
Higher (ABC1)	61 (78%)
Lower (C2DE)	14 (18%)
Unknown	3 (4%)
Motivation to process NHCs ^2^	
Mean (Standard Deviation)	3.24 (0.83)
Health or weight issues	
Overweight/obesity	20 (26%)
Irritable Bowel Syndrome or other digestive problems	14 (18%)
High blood cholesterol levels	7 (9%)
Hypertension	5 (6%)
Cancers (any type)	2 (3%)
Cardiovascular/heart disease	1 (1%)
Other chronic conditions/diseases	4 (5%)
Current diet status	
Self-chosen slimming diet	10 (13%)
Slimming diet prescribed by a health professional	3 (4%)
Cholesterol lowering diet	1 (1%)
Diabetic diet	1 (1%)
Other medical diet	2 (3%)

^1^ Socioeconomic status based on occupation status of the highest income earner in the household. Higher (ABC1) = higher and intermediate managerial or professional occupations, lower (C2DE) = unskilled, semi-skilled, skilled occupations and those unemployed. ^2^ Mean of three items on a scale from 1 to 5, with higher score indicating greater motivation to process NHCs.

**Table 2:** Overview of materials used in focus groups.

Product	Format	Claim(s) Displayed
Chocolate bar	Physical product	Regulated claim “no added sugar” as well as the claims “Good chocolate”, “Gluten & nut free”, “With live cultures”, “Over a billion lactobacillus & bifidobacterium”, and “63 calories per bar”
Breakfast cereal	Physical product	Regulated claims “Low in fat” and “Source of vitamin D”
Yoghurt	Photograph still from TV advertisement	Regulated claim “… contains calcium which helps maintain healthy bones”

**Table 3:** Overview of topic guide used in focus groups.

Section	Question/Topics
Introduction	Explanation of purpose and format of focus groups
	Ice-breaker e.g., favourite foods
General thoughts on food packaging	What do you think should be displayed on food packaging?
	Are there certain foods for which you are more likely to look at the label?
Rapid elicitation	Participants asked for quick initial thoughts on chocolate bar
Perceptions of products with nutrition claims (chocolate bar followed by breakfast cereal)	Do you think this product would taste good or taste bad?
	Do you think this product is healthy or unhealthy?
	Do you think this product would “fill you up” or leave you hungry?
Awareness of nutrition claims	Can you give me examples of nutrition claims?
	On which types of products are nutrition claims typically displayed?
Knowledge of nutrition claims	What does “low in fat” mean?
	Are there any advantages or disadvantages to having nutrition claims displayed on products?
Use of nutrition claims	Do you look for nutrition claims on packaging before eating a product?
	Has there ever been an occasion where a nutrition claim on a product has: stopped you from eating a product? Made you eat more of a product? Made you eat less of a product?
	How believable is this claim?
Perceptions of products with health claims (still from yoghurt advertisement)	Do you think this product would taste good or taste bad?
	Do you think this product is healthy or unhealthy?
	Do you think this product would “fill you up” or leave you hungry?
Awareness of health claims	Can you give me examples of health claims?
	On which types of products are health claims typically displayed?
Knowledge of health claims	What does “… contains calcium which helps maintain healthy bones” mean?
	Are there any advantages or disadvantages to having health claims displayed on products?
Use of health claims	Do you look for health claims on packaging before eating a product?
	Has there ever been an occasion where a health claim on a product has: stopped you from eating a product? Made you eat more of a product? Made you eat less of a product?
	How believable is this claim?
Finish	Do you have anything further about nutrition and health claims that we have not mentioned today that you would like to add?
	Summarise and clarify key points from discussion
